# Analysis of an Integrated Solar Combined Cycle with Recuperative Gas Turbine and Double Recuperative and Double Expansion Propane Cycle

**DOI:** 10.3390/e22040476

**Published:** 2020-04-21

**Authors:** Antonio Rovira, Rubén Abbas, Marta Muñoz, Andrés Sebastián

**Affiliations:** 1 Departamento de Ingeniería Energética, Universidad Nacional de Educación a Distancia (UNED), 28040 Madrid, Spain; mmunoz@ind.uned.es; 2 Departamento de Ingeniería Energética, Universidad Politécnica de Madrid (UPM), 28006 Madrid, Spain; ruben.abbas@upm.es (R.A.); andres.sebastian@upm.es (A.S.)

**Keywords:** ISCC, hybridization, concentrating solar power (CSP), advanced thermodynamic cycles, recuperative gas turbine, recuperative and double expansion (RDE) cycle

## Abstract

The main objective of this paper is to present and analyze an innovative configuration of integrated solar combined cycle (ISCC). As novelties, the plant includes a recuperative gas turbine and the conventional bottoming Rankine cycle is replaced by a recently developed double recuperative double expansion (DRDE) cycle. The configuration results in a fuel saving in the combustion chamber at the expense of a decreased exhaust gas temperature, which is just adequate to feed the DRDE cycle that uses propane as the working fluid. The solar contribution comes from a solar field of parabolic trough collectors, with oil as the heat transfer fluid. The optimum integration point for the solar contribution is addressed. The performance of the proposed ISCC-R-DRDE design conditions and off-design operation was assessed (daily and yearly) at two different locations. All results were compared to those obtained under the same conditions by a conventional ISCC, as well as similar configurations without solar integration. The proposed configuration obtains a lower heat rate on a yearly basis in the studied locations and lower levelized cost of energy (LCOE) than that of the ISCC, which indicates that such a configuration could become a promising technology.

## 1. Introduction

In the medium and long term, concentrating solar power (CSP) plants are going to be installed within grids that already include electricity generation by conventional thermal power plants. In this scenario, the implementation of integrated solar combined cycle (ISCC) plants leads to a rational and synergistic use of solar and fossil fuel resources.

ISCCs were initially proposed by Luz Solar International [1]. The technology is based on the use of parabolic trough collectors (PTC) that heat up a heat transfer fluid (HTF), which is usually a thermal oil. Solar energy contributes to increased steam production in a conventional combined cycle (CC) based on a gas turbine (GT) and a steam cycle coupled through a heat recovery steam generator (HRSG).

Most of the currently installed ISCC power plants follow the layout described above [2]. In the last decade, interest in ISCC has increased, and several aspects have been studied such as how to integrate the solar contribution into the combined cycle [3,4,5] or which technology is the best for collecting solar energy [6,7]. In most of these cases, the conceptual layout of the ISCC is preserved.

In addition to these kind of studies, there are also works that have proposed integrating solar into the CC using unconventional approaches that have substantially changed the ISCC layout. The main actor in these cases has been the thermodynamic cycle, which should be selected and optimized to simultaneously allow heat recovery and solar integration at adequate temperature levels.

Heat recovery from the exhaust gas of a GT and a solar energy supply from a HTF share the feature of being a finite or sensible type (heat transfer conveys a temperature change in the HTF). Another common feature is the maximum working temperature that each can reach, which is limited up to 400 °C or 500 °C. However, the temperature range is different, since waste energy from the GT exhaust gas must be recovered as much as possible while the HTF should maintain a certain temperature at the PTC inlet.

Organic Rankine cycles (ORCs) stand out among innovations for heat recovery from an exhaust gas. There are many works dealing with ORC coupled to a GT. Chacargetegui et al. [8] presented one of the first works configuring a CC using GT and ORC as the bottoming cycle. In a subsequent work [9], different strategies for the off-design operation were analyzed, using data from a commercial GT and including simple and recuperative GTs. Within the studied fluids for the ORC, toluene was the best candidate. An interesting finding was their conclusion that the use of recuperative GT increased the efficiency of the CC. Similarly, Cao et al. [10,11] compared the coupling of an ORC to a small GT, with both the GT and the ORC being recuperative, and concluded that, when the GT is recuperative, the configuration based on ORC enhanced the performance as compared with conventional steam turbines.

From these works, for fossil-fueled applications, it can be concluded that CC using recuperative GT and ORC as the bottoming cycle constitutes a good solution that increases the thermal efficiency of the CC.

Regarding solar-fueled CCs, Zare and Hasanzadeh [12] studied a configuration with two ORC cycles that replaced the conventional Rankine cycle, one cycle was dedicated to the intermediate cooling of a recuperative GT and the other cycle was coupled to the GT outlet. The gas turbine was fed by solar energy concentrated onto a central tower receiver, where air temperature reached values up to 900 °C. This configuration led to low exhaust gas temperatures, at ranges where ORCs were suitable. The fluid for the ORC was R123. Due to the increased interest in supercritical power cycles [13], Mohammadi, K. and McGowan [14] studied a similar system but installed a supercritical CO_2_ (sCO_2_) cycle as the bottoming cycle, where the gas turbine also included reheating, in addition to intercooling and recuperation. The authors compared toluene and isobutene for the ORC and concluded that isobutene had a better performance.

In the technical literature, there are many works that have studied innovative cycles for this range of temperature applications. Javanshir et al. [15] and Rovira et al. [16] focused on the comparison of thermodynamic cycles for CSP applications with low maximum temperatures (up to 400 °C), including ORC and other advanced cycles. These studies did not consider any topping GT, and thus combined cycles were not regarded. Likewise, Petrollese and Cocco [17,18] also evaluated a recuperative ORC driven by concentrated solar energy. Some solutions involving innovative combined cycles have been proposed, such as the case of works by [19,20,21], where the topping cycle was a sCO_2_ and the bottoming cycle was an ORC.

Recently, the hybrid Rankine and Brayton (HRB) cycle [22,23] and double recuperative and double expansion (DRDE) cycle [24] have been proposed for working with finite sources at maximum temperatures of 400 °C. While the HRB cycle is suitable for closed heat sources [24], DRDE is a good solution for heat recovery. Due to the range of working temperatures, the latter cycle is a good candidate to be installed as the bottoming cycle of CC using a recuperative GT.

The present work intends to take a further step in the optimization of ISCCs by means of introducing the previously developed DRDE cycle as the bottoming cycle. This cycle replaces the conventional steam Rankine cycle and, due to its maximum temperature limitation, a recuperative GT is included instead of a conventional one. The DRDE cycle and its integration within the ISCC are analyzed and optimized. The performance of the proposed ISCC is assessed at design conditions and at off-design operation (daily and yearly). All results are compared to those obtained under the same conditions by a conventional ISCC.

Both reference and proposed cycles are described in Section 2. Then, Section 3 introduces the methodology and the merit numbers. Section 4 presents the results. Finally, Section 5 is devoted to the conclusions.

## 2. Configurations

This section presents the different power cycle configurations studied in this work. First, a conventional combined cycle and a conventional integrated solar combined cycle are defined for comparative purposes. Then, the novel double recuperative and double expansion cycle, used as the bottoming cycle, is depicted along with its integration into both reference power plants.

### 2.1. Conventional CC

**Figure 1:**
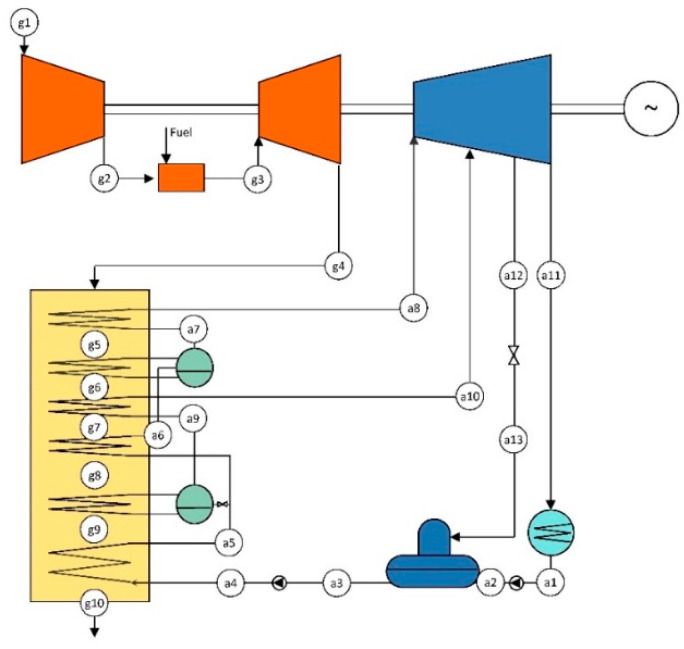
Layout of the reference combined cycle gas turbine (CCGT).

A conventional CC without solar contribution, namely the combined cycle gas turbine (CCGT), is considered as the reference power plant. The exhaust gas of the GT is directed to a dual pressure-level HRSG. The steam that is generated drives the steam turbine (ST) of the Rankine cycle. Table 1 presents the design parameters, which lead to a nominal power rate of about 125 MW. Figure 1 shows the layout of the configuration, where HP and LP refer to high- and low-pressure levels, respectively.

### 2.2. Conventional ISCC

**Figure 2:**
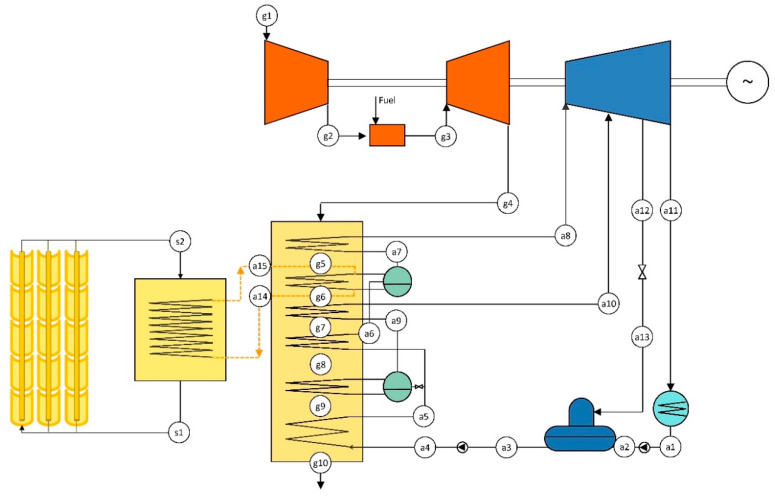
Layout of the reference integrated solar combined cycle (ISCC).

For comparative purposes, the definition of a reference ISCC configuration is required. This ISCC should be based on the CCGT configuration. Thus, it uses a conventional GT and a dual-pressure level HRSG that feeds the steam turbine. Additionally, the ISCC includes a 16 MW_th_ PTC solar field that heats a thermal oil. Then, the thermal oil is directed to a solar steam generator (SSG) to evaporate water at the high-pressure level of the HRSG. Therefore, the SSG works in parallel with the high-pressure evaporator of the heat recovery steam generator. The configuration is depicted in Figure 2. Design parameters are presented in previous Table 1. Table 2 shows the data of the PTC solar field.

The solar integration results in increased steam generation as compared with the CCGT configuration. Therefore, the nominal power rate of the ISCC is higher than that of the CCGT, reaching 130 MW.

### 2.3. CCGT-R-DRDE and ISCC-R-DRDE Configurations

As mentioned previously in the Introduction, an innovative proposal is presented which involves the use of a recuperative GT and the DRDE cycle as the bottoming cycle. For the sake of a fair comparison, a reference CC without solar integration but one that includes a recuperative GT and a DRDE cycle is required.

**Figure 3:**
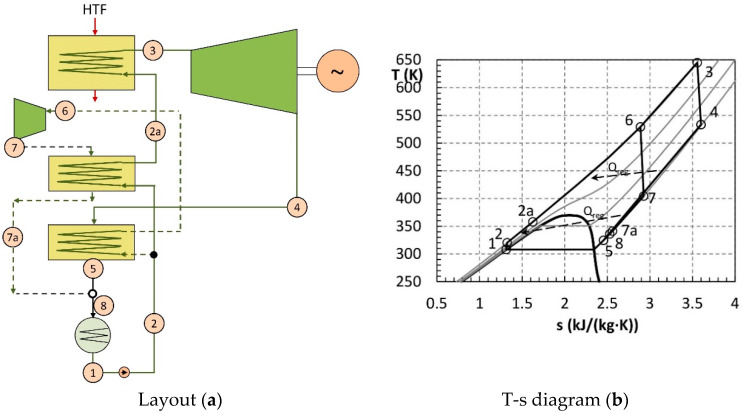
Layout of the double recuperative double expansion (DRDE) cycle (**a**) and its T-s diagram (**b**) [24].

A detailed description of the DRDE cycle is found in [24], which includes its comparison to other thermodynamic cycles. Briefly, the DRDE cycle consists of an ORC-like cycle with two parallel heating lines working at a supercritical pressure. The main one is fed by the heat source (in the present work, the heat recovery from the GT exhaust gas), where it is heated from 2a to 3 in Figure 3, and the generated vapor is expanded in the main turbine (*VT_main_*). The second heating line is heated (from 2 to 6) in a recuperator fed by steam coming from the outlet of the main turbine. The vapor generated in the recuperator is expanded in a secondary turbine (*VT_secondary_*). Finally, the vapor at the exit of the secondary turbine is directed to a secondary recuperator, which slightly preheats the fluid of the main heating line (from 2 to 2a in Figure 3). The scheme of the cycle and the temperature–entropy (*T*-*s*) diagram are shown in Figure 3.

Propane is a possible working fluid that fits well with the nature of the cycle (regarding the critical temperature, pressure, and slope of the saturated vapor in the T-s diagram) [24].

**Figure 4:**
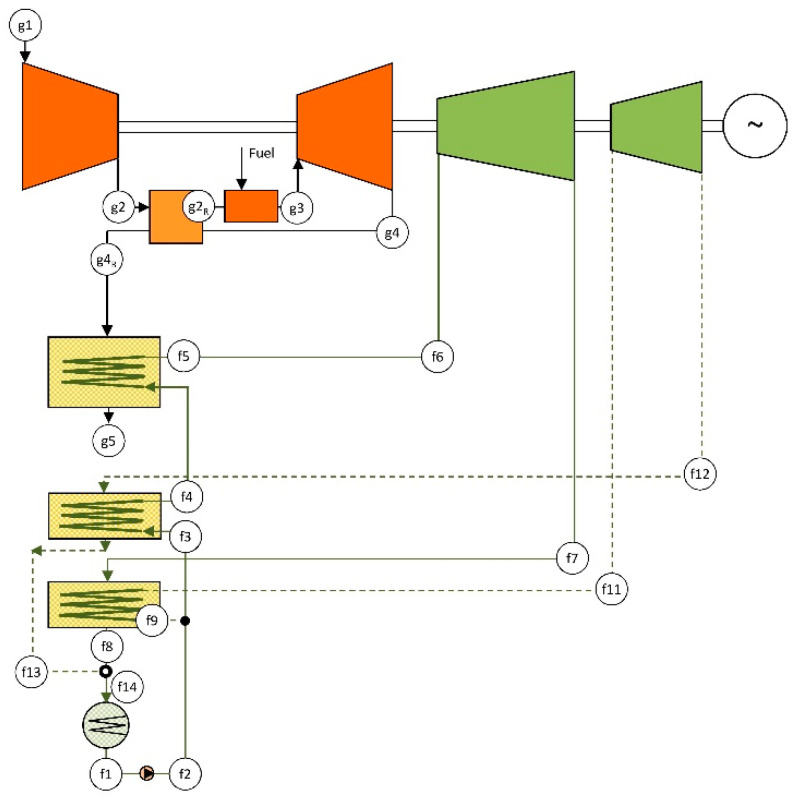
Layout of the reference CCGT-R-DRDE.

The combined cycle using recuperative GT and DRDE cycle, namely CCGT-R-DRDE, consists of the same gas turbine as in the CCGT configuration but includes a recuperator, and the DRDE cycle is placed as the bottoming cycle, coupled through a heat recovery vapor generator (HRVG). The use of a recuperative GT is advisable due to the working temperature limitation of propane, which is set at 370 °C. The design parameters are shown in Table 1, and the layout is shown in Figure 4.

The main difference between the gas turbine in the CCGT and CCGT-R-DRDE configurations is that, in the latter, the exhaust gas is directed to the recuperator, and therefore the air exiting the compressor is preheated. This results in a fuel savings in the combustion chamber at the expense of a temperature decrease of the exhaust gas. Thus, the waste energy available for generating steam is lower, which conveys a decreased power rate for the bottoming cycle, and subsequently, for the whole combined cycle, resulting in 109 MW.

A further power rate decrease in the gas turbine is expected due to the additional pressure drop that the recuperator introduces on the air side, which should be considered. On the gas side, the total pressure drop from the turbine outlet to the environment is considered to be unchanged (regarding the other configurations) since the additional pressure drop caused by the recuperator is partially mitigated in the HRVG, as the decrease of heat recovery conveys a heat exchange area decrease.

In order to integrate solar energy into this configuration, a solar field is added to the layout above, leading to an ISCC-R-DRDE configuration. The considered solar field is analogous to that described in a previous section, involving PTC that heats up a thermal oil (see features in Table 2). The thermal oil is directed to a solar vapor generator (SVG) that generates additional propane vapor.

The first decision for the optimization of the solar integration is to select the integration point for the solar contribution. It can be placed either at the main heating line (line of points from 2 to 3 in Figure 3) or at the secondary one (line of points from 2 to 6 in Figure 3). In order to reach high solar-to-mechanical efficiency conversion rates, the propane mean temperature along the heating process must be high.

In the case of selecting the main heating line, as the temperature at the beginning of the line is low (propane is pumped from the condenser and slightly preheated at the secondary recuperator, point 2a in Figure 3), first, the fluid should be preheated by the exhaust gas in the HRVG, and then split into two streams, one that finalizes the heating process in the HRVG and the other that goes to the SVG. Thus, the HRVG must have two heat exchangers, one devoted to preheating and a second heater to provide the maximum temperature to only a fraction of the fluid at nominal conditions. At low or null solar irradiation conditions, the preheater and the heater work with the same mass flow rate because the SVG is inactive. This makes the heat recovery very effective due to the supercritical state of propane. However, at high irradiation conditions, the mass flow rate in the heater would be lower than in the preheater. This should lead to inefficiency in the heat recovery process because of the low energy recovery at the heater. For these reasons, this integration choice was discarded.

**Figure 5:**
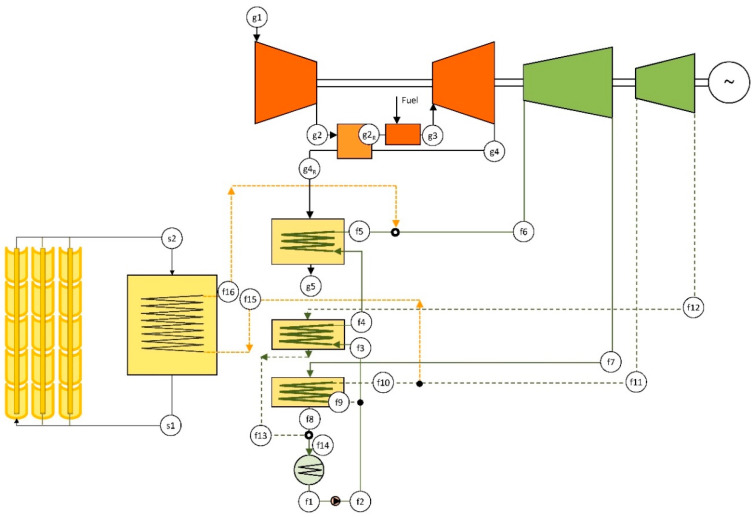
Layout of the reference ISCC-R-DRDE.

Another choice, illustrated in Figure 5, is to integrate the solar contribution in the secondary heating line. In this way, the optimal point to supply the solar energy takes a fraction of the propane at the exit of the main recuperator (point f10 in Figure 5). Then, the propane removed from the secondary heating line is heated to the maximum temperature in the SVG and, finally, added to the main heating line (point f5 in Figure 5). As a result, the heat recovery in the HRVG is not affected by the solar irradiation conditions, which should lead to low inefficiency regardless of the operating conditions.

As can be observed, the integration point for the ISCC-R-DRDE configuration is different from that of the conventional ISCC configuration. In the latter, solar integration is destinated to evaporate a fraction of the high-pressure level steam, reducing the contribution of the high-pressure evaporator. Due to the fact that the high-pressure evaporator is the heat exchanger with the highest irreversibility, solar integration contributes to an increase of the exergy efficiency. In the case of the ISCC-R-DRDE configuration, due to the nature of the supercritical propane, the HRVG is very effective and irreversibility is low. For that reason, there is no room for synergistic improvements due to solar integration and the best solution from an exergy point of view is to set the integration point in the secondary heating line and maintain the heat recovery unaffected.

## 3. Methodology

The methodology followed in this work is conceived to carry out a consistent comparison among the proposed and the reference power cycles in order to assess the potential benefits of DRDE configuration as the bottoming cycle. First, the simulation process at nominal conditions along with the main assumptions for this analysis is presented. Then, off-design performance is modeled to account for daily and yearly variations. Indeed, annual performance methodology is set in the third subsection. Finally, merit numbers and key parameters are described.

### 3.1. Simulation at Nominal Conditions

For the simulation of the different subsystems and equipment, mass and energy balances are calculated. In addition, some parameters are required, either technological or design ones, in order to establish the power plant nominal conditions.

The compressor of the gas turbine compresses air from nearly ambient pressure (there is a pressure drop at the compressor inlet that is set to 20 mbar) up to a pressure given by the pressure ratio (*r*). Then, the air is directed to the combustion chamber (CCGT and ISCC configurations) or to the recuperator (CCGT-R-DRDE and ISCC-R-DRDE ones). For the compression process, a polytropic efficiency of 90% is considered.

The GT recuperator is simulated through the energy balance and its effectiveness (*ε*), which relates the actual air temperature increase to the maximum temperature available:

(1) $$\varepsilon =\frac{T_{g2R}-T_{g2}}{T_{g4}-T_{g2}},$$

where points are referred to Figure 4 or Figure 5. Effectiveness at nominal operation takes the value of 80%. Pressure drop (*ξ_R,GT_*), at the air side, is calculated as below [25]:

(2) $$\xi_{R,gt}=\left(\varepsilon -0.48\right)/30.$$

A pressure drop of 5% is considered in the combustion chamber.

The gas exiting the combustion chamber is directed to the turbine, where it is expanded considering a polytropic efficiency of 90%. A back pressure of 40 mbar is considered due to pressure drops in the HRSG or HRVG and the GT recuperator.

Thermophysical properties for air and exhaust gas are taken from [26].

For the HRSG (CCGT and ISCC configurations), the heat balances for economizers, evaporators, and superheaters are required. Additionally, the steam temperature and pressure, pinch points, approach points, and pressure levels should be selected (Table 1). The deaerator pressure is set to 0.2 bar, and it is fed by steam coming from the steam turbine at 1.2 bar that is previously laminated. Therefore, the feedwater temperature at the HRSG inlet is about 60 °C.

Likewise, for the HRVG (CGGT-R-DRDE and ISCC-R-DRDE configurations), the heat balance to the corresponding heat exchanger is required. The maximum temperature of propane is set at 370 °C and the working pressure is 170 bar. The DRDE cycle simulation also requires heat balances for the main and secondary recuperators and, with respect to this, a pressure drop of 2% is considered. The pinch point is set at 10 °C.

Steam and vapor turbines are simulated considering an isentropic efficiency of 85%. The working pressure of the condenser corresponds to a saturation temperature of 35 °C, i.e., 65 mbar for water and 12 bar for propane. The pump’s efficiency is set to 75%. Nominal ambient temperature is 15 °C.

Water-steam properties were taken from [27] and the propane properties were taken from [28].

For the SSG and SVG, the pinch points are set at 10 °C. The selected thermal oil is Therminol VP1. Properties are taken from [29], and the maximum working temperature is limited to 390 °C. The minimum temperature for the HTF is set by the pinch point (10 °C) and the temperature of the heating fluid at the heat exchanger inlet, i.e., saturated water coming from the high-pressure drum or propane coming from the secondary recuperator.

The solar field considered consists of several loops of a set of PTCs in series. The considered trough design is Eurotrough, whose features are shown in Table 2. The simulation of the solar field takes into account the solar energy collected and concentrated, as well as the heat transferred to the fluid and the corresponding losses, both optical errors and thermal losses. Convection losses are calculated using Petukov’s correlation, and Colebrook’s equation is used to estimate the pressure drop inside the troughs.

A nominal direct normal irradiation (DNI) of 850 W/m^2^ is considered. Provided the nominal mass flow rate recommended inside the troughs (Table 2), the local PTC efficiency (*η_PTC,l_*) can be estimated with the following expression [30]:

(3) $$\eta_{PTC,l}=-1.3\cdot 10^{-6}\cdot T^{2}\;\left({}^{\circ}C\right)+3.13\cdot 10^{-4}\cdot T\;\left({}^{\circ}C\right)+0.69563.$$

As the HTF temperature at the inlet and outlet of each loop is known, the length required for the loop can be obtained integrating the equation below:

(4) $$DNI\cdot IAM\cdot W_{PTC}\cdot \eta_{PTC,l}\;\left(T\right)\cdot dL={\dot{m}}_{HTF,loop,des}\cdot c_{p}\cdot dT,$$

where *W* is the PTC width and *L* the loop length.

The obtained length must be decreased in order to obtain a multiple of the single collector length. This decrease leads to an actual mass flow rate slightly smaller than the reference of Table 1. Then, the actual energy gain per loop can be calculated. The number of considered loops depends on the desired solar thermal contribution, which results in 16 MW_th_ for the ISCC configuration (11 loops of 39 modules) and 15 MW_th_ for the ISCC-R-DRDE one (8 loops of 50 modules).

### 3.2. Simulation at Off-Design Operation

Off-design operation takes place when either ambient conditions are different from the nominal ones or in scenarios of part-load operation. In the present work, the simulation for the daily and yearly operation is done assuming maximum energy dispatching. Thus, the gas turbine works at full load all of the time and off-design operation is due to the variation of ambient conditions.

The off-design operation behavior of the compressor is determined by its characteristic curves. For a given compressor working with fixed blade geometry, the characteristic curves relate compressor pressure ratio and efficiency to mass flow rate and shaft speed. In this work, these curves are based on [31]. Simulation of the combustion chamber is carried out as at nominal operation by means of the mass and energy balances. As the GT turbine works at full load, turbine inlet temperature is kept constant. Analogously to the compressor, simulation of the GT turbine is done using characteristic curves. In this case, correlations from [32] are used.

The off-design operation of the heat exchangers of recuperators, HRSG, HRVG, SSG, and SVG is calculated using the corresponding heat balances and the heat exchange equation:

(5) $$\dot{Q}=UA\cdot \frac{\Delta T_{hot-end}-\Delta T_{cold-end}}{ln\left(\Delta T_{hot-end}/\Delta T_{cold-end}\right)},$$

where $\dot{Q}$ is the thermal power exchange and *UA* is thermal conductance.

The *UA* product variation is evaluated as below [33]:

(6) $$U/U_{design}={\left(\frac{\dot{m}}{{\dot{m}}_{design}}\right)}^{q},$$

where *ṁ* corresponds to the stream with the highest heat transfer resistance and *q* takes the value of 0.625 for Prandtl numbers of roughly 0.7 (HRSG, HRVG, and recuperators) and 0.8 for Prandtl numbers from 5 to 7 (SSG and SVG).

Regarding steam turbines, the off-design capacity is evaluated using the Stodola–Frügel law or ellipse law:

(7) $$\dot{m}\cdot \sqrt{\frac{T_{inlet}}{p_{inlet}^{2}-p_{outlet}^{2}}}=constant,$$

that can be used for each turbine cylinder or section with constant mass flow rate (without intermediate bleedings). The variation of isentropic efficiency is assessed using the correlation proposed in [34]. This correlation introduces a decrease of 1 percentage points in the isentropic efficiency for each 3 points of turbine capacity $\left(\dot{m}\cdot \sqrt{T_{inlet}}/p_{inlet}\right)$ decrease.

For the condenser, the saturation temperature variation is estimated as half of the ambient temperature variation and the condensation pressure is calculated accordingly.

Finally, regarding the solar field, the PTC efficiency is obtained applying the heat balances and considering a DNI corrected by the incidence angle [35]. The temperature of Therminol VP1 is fixed at 390 °C at every condition. For that, the oil mass flow should be varied. A DNI threshold of 300 W/m^2^ is required to ensure a correct collector cooling.

### 3.3. Annual Performance

Almeria and Las Vegas were the sites selected for the analyses of the proposed configurations [7]. A typical meteorological year (TMY) was used for both sites. The calculation step was 1 h. A previous analysis of the TMY was completed before running the simulations in order to reduce the calculating points as much as possible. Instead of using 8760 points per year, the analysis resulted in the determination of a frequency matrix that related each ambient condition to the total yearly hours that such a condition takes place within the TMY. The analysis led to 538 operating conditions in Almeria and 909 in Las Vegas. An example of these matrices is found in [7].

### 3.4. Merit Numbers

Yearly energy generation is evaluated as the summation of the product of yearly frequency (of each operating condition), *n*, by power rate (at this condition), *P*. Yearly averaged thermal efficiency can be calculated by the following equation:

(8) $$\eta =\frac{\sum^{\text{​}}n\cdot t\cdot \left(P_{GT}+P_{ST}\right)}{\sum^{\text{​}}n\cdot t\cdot \left({\dot{m}}_{f}\cdot H_{c}+{\dot{Q}}_{sol,\mathrm{gross}}\right)}.$$

In the above expression, *t* is one hour, *ṁ_f_* is the fuel mass flow, and *H_c_* is the lower heating value of the fuel (natural gas, 48.000 kJ/kg).

In hybrid systems with two thermal sources, the use of thermal efficiency can hide relevant conclusions. In fact, in ISCCs, it has two drawbacks. First, the so-defined efficiency does not take into account the individual contribution of each source. Secondly, the solar energy contribution is penalized, since solar heat is integrated into the low-temperature cycle and is not congruent with the objective of the plant.

A conventional alternative for the assessment of solar contribution in ISCC (valid only for boosting strategy) is the use of the incremental solar-to-electricity efficiency [36], which relates the incremental production of the hybrid system to the supplied solar energy supplied. In this work, a more advanced efficiency defined in [37] is used, namely internal solar-to-electricity efficiency (*η_ise_*), which assesses the individual contribution of each heat source according to the irreversibility associated with each heat source:

(9) $$\eta_{ise}=\frac{E_{ise}}{\sum^{\text{​}}n\cdot t\cdot {\dot{Q}}_{sol,gross}}$$

where *E_ise_* is:

(10) $$E_{ise}=\sum^{\text{​}}\left(\frac{n\cdot t\cdot \left(P_{gt}+P_{st}\right)\cdot {\dot{E}}_{PTC\to cycle}}{{\dot{E}}_{f\to cycle}+{\dot{E}}_{PTC\to cycle}}\right).$$

Note that the above efficiency can refer to either gross or net solar energy, which are related through the PTC efficiency.

In addition to the efficiencies defined above, the complete power plant behavior is analyzed using the heat rate (HR) and the levelized cost of energy (*LCOE*):

(11) $$HR=\frac{\sum^{\text{​}}n\cdot t\cdot {\dot{m}}_{f}\cdot H_{c}}{\sum^{\text{​}}n\cdot t\cdot \left(P_{gt}+P_{st}\right)};$$

(12) $$\;LCOE=\frac{LC_{Inv}+LC_{O\&M}+LC_{f}}{E_{yearly}}\;,$$

where *LC_inv_*, *LC_O&M_*, and *LC_f_* are the levelized cost of equipment acquisition, operating/maintenance and fuel, respectively, and *E_yearly_* is the yearly energy production. Economic parameters used for the LCOE calculation are shown in Table 3.

In order to economically assess the solar contribution, the internal solar-to-electricity cost (*C_ise_*) is used as follows:

(13) $$C_{ise}=\frac{{\left(LC_{Inv}+LC_{O\&M}\right)}_{ISCC}-{\left(LC_{Inv}+LC_{O\&M}\right)}_{CCGT}}{E_{ise}}.$$

## 4. Results and Discussion

The main results obtained for the configurations considered are now presented. These are divided into four subsections. First, both fossil-fueled power plants using the conventional steam cycle and the DRDE cycle as the bottoming cycle are assessed. Then, results for the conventional ISCC are examined. Third, the effect of implementing the DRDE cycle instead of the conventional steam cycle on an ISCC is shown. Finally, the daily and yearly performance of all configurations presented are compared.

### 4.1. Performance of Reference Configurations without Solar Contribution

In this section, the performances of the reference CCGT and the CCGT-R-DRDE configuration are presented, working at some representative operating conditions. The results are shown in Table 4. Data with the thermodynamic properties of the fluids and mass flow rates are attached as Supplementary Material.

First of all, the results show that CC technology is highly affected by ambient conditions. At a high ambient temperature, the density of air is low, which implies a low compressor mass flow and a low GT power rate. Lower air mass flow also conveys lower heat recovery in the HRSG and HRVG, although the power decrease is lower in the bottoming cycle than in the GT. Conversely, a low ambient temperature leads to a higher power rate for the GT and the bottoming cycle.

In addition, it is observed that the CCGT-R-DRDE configuration, which includes a recuperative gas turbine, reaches a lower power rate than that of the conventional CCGT. As commented in Section 2, this is due to the lower waste energy available for heat recovery, since part has been used to preheat the compressed air before the combustion chamber. However, the GT efficiency is higher for the CCGT-R-DRDE configuration thanks to the fuel saving.

Finally, one can observe that the CCGT-R-DRDE configuration reaches the best efficiency, due to the effect of the recuperative GT and because the waste thermal energy is very effectively recovered in the HRVG.

### 4.2. Performance of the Reference ISCC

Table 5 presents the results for the ISCC configuration in the same representative operating points as in previous section (thermodynamic properties and mass flow rates can be found in the Supplementary Material). In this case, due to the solar contribution, two different solar irradiation conditions were considered for each ambient temperature.

It is important to note that the ISCC configuration requires a larger steam turbine than the CCGT, since it should work with a higher steam mass flow rate due to the solar contribution. This fact affects the performance at low or null solar irradiations, particularly through the steam working pressure, because the steam turbine is oversized for those conditions. For the same reasons, all HRSG heat exchangers at a high-pressure level, except for the evaporator, are larger for the ISCC configuration than that of the CCGT. The higher heat exchange area mitigates, to some extent, the performance decrease caused by using an oversized steam turbine.

At null solar irradiation, the ISCC reaches a power rate very similar to those obtained for the CCGT. As expected, the ISCC power is slightly lower due to the turbine oversizing, which leads to a lower working pressure and a lower isentropic efficiency for the steam turbine.

At high solar irradiation, the power rate of the ISCC increases significantly. Accordingly, the HR improves over those obtained for the CCGT, although the value of efficiency using the conventional definition decreases. Additionally, the steam working pressure reaches values close to the nominal values, because the steam mass flow value is also similar to the nominal value.

Solar-to-electricity efficiency takes values from 35% to 40% in net values (23% to 26% gross), and PTC efficiency is roughly 65%.

### 4.3. Performance of ISCC-R-DRDE

Table 6 shows the results for the ISCC-R-DRDE configuration at the same operating conditions as in previous sections (thermodynamic properties and mass flow rates are found in the Supplementary Material). As in the case of the ISCC, the turbines of the bottoming cycle are larger than those corresponding to the reference configuration, i.e., CCGT-R-DRDE, due to the solar contribution. Likewise, this oversizing affects the performance, decreasing the working pressure and isentropic efficiency at low or null solar irradiation.

At null solar irradiation, the power reached by the ISCC-R-DRDE is very similar to those corresponding to the CCGT-R-DRDE. At high solar irradiation, the power rate significantly increases, and HR improves. Similar to the ISCC case, the working pressure value is close to the nominal value.

The ISCC-R-DRDE presents a better performance than that of the ISCC, therefore the use of the DRDE cycle as the bottoming cycle together with a recuperative gas turbine is advisable from the thermodynamic point of view.

Regarding the efficiency of the solar contribution, the solar-to-electricity efficiency ranges from 35% to 39% in net values (22% to 25% in gross values). The PTC efficiency is roughly 65%.

### 4.4. Daily and Yearly Operation

Once the performance of the different configurations has been analyzed at various representative working conditions, their daily and yearly behaviors are studied. As commented in the Introduction, the power rate for the analysis is the maximum for each ambient temperature.

**Figure 6:**
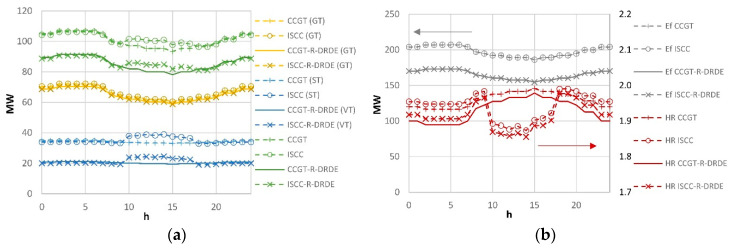
Daily energy production (**a**) and fuel consumption and heat rate (**b**) (Las Vegas June 27).

Figure 6 shows the results obtained on a typical summer day in Las Vegas (June 27 of the TMY), with a maximum DNI of roughly 900 W/m^2^. For all configurations, the maximum power is obtained during the morning due to the low ambient temperature. As expected, the power rate and fuel consumption (*E_f_*) of the configurations using recuperative GT are lower than those obtained for the non-recuperative configurations.

**Figure 7:**
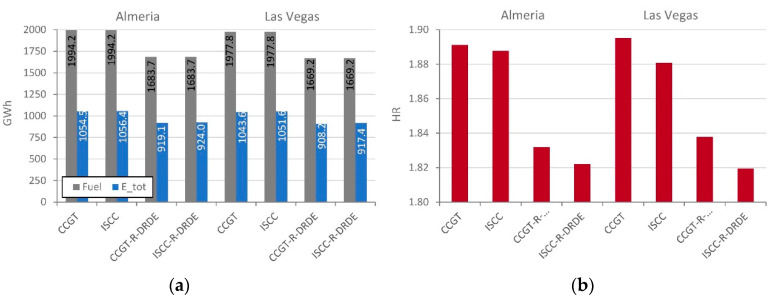
Yearly fuel consumption and energy production (**a**) and HR (**b**).

Figure 7a shows the yearly production (*E_tot_*) and fuel consumption for all configurations in Almeria and Las Vegas. It can be observed that configurations with recuperative GT and DRDE bottoming cycle require lower yearly fuel consumption than that of the non-recuperative GTs, although they generate lower energy. Figure 7b shows the heat rate (that relates fuel consumption to energy production). It can be observed that the heat rate is lower for the configurations based on the DRDE cycle. Thus, they are advisable from a thermodynamic perspective. In addition, one can observe that solar integration leads to lower heat rates in Las Vegas than in Almeria thanks to the higher annual irradiation, whereas combined cycles with no solar integration achieve lower heat rates in Almeria due to lower mean temperatures.

As expected, HR is lower for the recuperative configurations due to the lower fuel consumption. Additionally, solar integration helps to reduce the HR, even more obviously in Las Vegas. Finally, the ISCC-R-DRDE reaches the best result, which improves the performance over the conventional ISCC.

**Figure 8:**
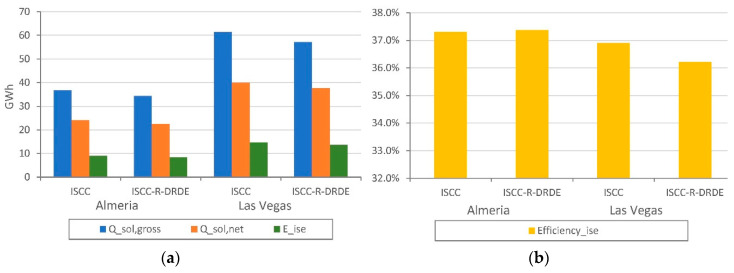
Yearly solar contribution (**a**) and solar-to-electricity efficiency (**b**).

Regarding the solar contribution, Figure 8a shows the solar energy supplied to the plants in gross, net, and internal solar-to-electricity terms. It is important to highlight that the solar contribution is much higher in Las Vegas than in Almeria, since ambient conditions are much more favorable (higher DNI) for CSP despite the similar latitude of both sites. Although the solar contribution is very similar in all cases, the conventional ISCC reaches the maximum contribution while the ISCC-R-DRDE reaches the minimum contribution. It is noted that the gross solar energy supplied is not exactly the same for ISCC and ISCC-R-DRDE in the same location due to the slightly different number of solar loops used.

Figure 8b shows the internal solar-to-electricity efficiency for all configurations, which reaches values above 35% in all cases, regardless of the site. Similar to the case of the solar contribution, internal solar-to-electricity efficiencies are very similar in all cases. One can observe that the ISCC tends to reach a higher internal solar-to-electricity efficiency than that of the ISCC-R-DRDE, as the ST efficiency is higher than the VT cycle. This is due to the higher temperature of the exhaust gas contribution.

**Figure 9:**
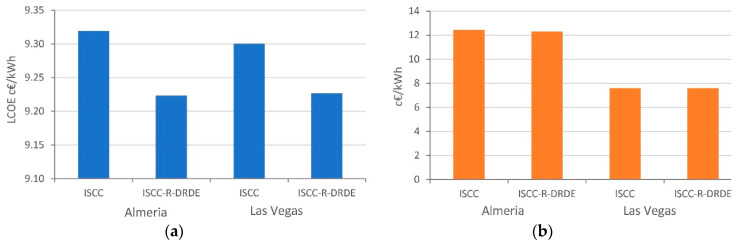
Lower levelized cost of energy (LCOE) (**a**) and solar-to-electricity incremental costs (**b**).

Finally, Figure 9 shows the LCOE and the internal solar-to-electricity costs. It is observed that the ISCC-R-DRDE configuration reaches a lower LCOE than ISCC in the proposed scenario of limited power, which indicates that such a configuration could become a promising technology. Regarding the solar-to-electricity cost, one can observe that it is lower in Las Vegas than in Almeria, due to the more favorable conditions. Under the proposed scenario, the cost obtained for the ISCC-R-DRDE configuration is lower in Almeria and the same as the conventional ISCC in Las Vegas. Nevertheless, cost models for this configuration introduce higher uncertainties than in other ones, as they involve technology and equipment less developed. Therefore, the results must be understood from the point of view of being an interesting choice that could lead to promising results.

## 5. Conclusions

A new configuration for combined cycles with or without solar integrations has been presented. The novelty of these configurations is the use of a recuperative gas turbine and an innovative organic bottoming cycle, the double recuperative double expansion cycle (DRDE). The use of recuperative gas turbines seeks fuel saving, but also leads to lower temperatures at the outlet (around 130 °C reduction), and thus lower sizes of the bottoming cycle.

One can conclude that both configurations, CCGT-R-DRDR and ISCC-R-DRDE, achieve important efficiency improvements as compared with the state-of-the-art CCGT and ISCC. However, the fuel saving is more important for cold ambient temperatures (around 4%) than for high temperatures (around 2.5%), whereas it does not vary with the solar irradiance level.

Two case studies were compared, i.e., Almeria and Las Vegas; the former has a lower mean ambient temperature and for the latter the annual irradiation is notably higher. As a result, non-hybridized technologies achieve lower values of heat rate in Almeria, whereas integrated solar combined cycles minimize the heat rate in Las Vegas. Nevertheless, in both locations innovative configurations achieve an important heat rate reduction. The annual mean heat rates for both CCGT-R-DRDE and ISCC-R-DRDE are between 1.82 and 1.84 in both locations, whereas the state-of-the-art CCGT and ISCC lead to values between 1.88 and 1.90. Therefore, one can conclude that, from the energetic perspective, the proposed configurations are very interesting.

An economic assessment was also carried out. Although uncertainties are very high for new technologies that require components that have not been developed, this assessment concludes that the ISCC-R-DRDE achieves similar values for the LCOE than that of the state-of-the-art ISCC plants, or even slightly lower.

The development of the required components such as high-pressure propane turbine and heat exchangers should be considered in the near future in order to assess the feasibility of the proposed cycles from a technological perspective.

## Figures and Tables

**Table 1:** Design parameters of the different configurations.

**Gas Turbine**	
**Ambient conditions**	15 °C, 1 bar
Compressor pressure ratio	16:1
Air mass flow rate	210 kg/s
Turbine inlet temperature	1227 °C
Efficiency of combustion chamber	95%
**Steam Cycle HRSG**	
Steam temperature	545 °C
HP/LP pressure	90 bar/5 bar
Pinch points	10 °C
Approach points	20 °C
LP temperature difference	10 °C
**DRDE Cycle HRVG**	
Vapor temperature	370 °C
Pressure	170 bar
Pinch point	10 °C
Mechanical efficiency	98%

**Table 2:** Features of the parabolic trough collectors.

Subject	Data
Outer and inner tube diameter	0.07/0.065 m
Outer and inner glass envelope diameter	0.115/0.109 m
Module and mirror length	12.27/11.9 m
Intercept factor	92%
Reflectivity of mirrors	92%
Transmissivity of glass	94.5%
Absorptivity of tubes	94%
Optical efficiency (peak)	75%
Thermal emissivity	4.795·10^−2^ + 2.331·10^−4^·T (°C)
Maximum oil mass flow rate recommended	7.725 kg/s

**Table 3:** Economic data.

Subject	Cost
PTC cost	200 €/m^2^
Land cost	2 €/m^2^
Specific cost for the power block [34,38]	(466.1 + 113900/*P*[MW]) €/kW
Steam turbine cost variation [39]	(0.207·Δ*P*[MW]) M€
Gas turbine recuperator * [40]	(2861·*A*^0.59^[m^2^]) $
SSG^+^ [3]	(−7·10^−4^·*A*^2^[m^2^] + 126.9·*A*[m^2^] + 7770.4) €
Combined cycle O/M cost	17.9 €/(year·kW)
Solar field O/M cost	9 €/(year·m^2^)
Surcharge for construction, engineering, and contingencies	10%
O/M equipment cost percentage of investment	1%
Interest rate	4%
Fuel escalation rate	2.5%
O/M escalation rate	1%
Natural gas	0.0232 €/kWh
Life	25 years

* Convective heat transfer of 700 Wm^−2^K^−1^ [40] and ^+^ Convective heat transfer of 1500 Wm^−2^K^−1^ [41].

**Table 4:** Results for configurations without solar contribution.

Tamb (°C)	Configuration	*P_CC_* (MW)	*P_GT_* (MW)	*P_ST,VT_* (MW)	*HR*	*η*	*η_GT_*	*η_ST,VT_*	*T_exh,GT_* (°C)	*p_max_* (bar)
0	CCGT	137.7	99.0	38.7	1.85	54.1%	38.9%	34.7%	558	90.6
	CCGT-R-DRDE	122.1	96.9	25.2	1.78	56.3%	44.7%	29.5%	435	169.4
15	CCGT	124.8	87.7	37.1	1.88	53.2%	37.4%	34.0%	575	90
	CCGT-R-DRDE	109.3	85.8	23.5	1.82	55.1%	43.2%	28.4%	450	169.1
30	CCGT	109.0	73.8	35.2	1.93	51.9%	35.2%	33.3%	598	88.6
	CCGT-R-DRDE	93.5	72.1	21.4	1.88	53.2%	41.0%	27.3%	468	165.5

**Table 5:** Results for the ISCC configuration.

Tamb (°C)	Irradiation (W/m^2^)	*P_CC_* (MW)	*P_GT_* (MW)	*P_ST,VT_* (MW)	*ṁ_f_·H_c_* (MW)	*Q_sol,net_* (MW)	*η*	*HR*	*η_ise_*	*p_max_* (bar)
0	0	136.7	99.0	37.7	255	0	53.7%	1.86	-	75
15	0	123.9	87.7	36.2	234	0	52.9%	1.89	-	75
30	0	108.2	73.8	34.4	210	0	51.5%	1.94	-	73
0	850	142.7	99.0	44.1	255	16.1	52.8%	1.78	40.6%	91
15	850	130.1	87.7	42.4	234	16.1	52.0%	1.80	38.8%	90
30	850	114.4	73.8	40.5	210	16.1	50.6%	1.84	36.6%	89

**Table 6:** Results for the ISCC-R-DRDE configuration.

Tamb (°C)	Irradiation (W/m^2^)	*P_CC_* (MW)	*P_GT_* (MW)	*P_ST,VT_* (MW)	*ṁ_f_·H_c_* (MW)	*Q_sol,net_* (MW)	*η*	*HR*	*η_ise_*	*p_max_* (bar)
0	0	121.9	96.9	25.0	217	0	56.2%	1.78	-	117
15	0	108.9	85.8	23.0	198	0	54.8%	1.82	-	116
30	0	92.7	72.1	20.6	176	0	52.8%	1.90	-	114
0	850	126.9	96.9	30.7	217	15.3	54.9%	1.70	38.9%	161
15	850	114.4	85.8	28.6	198	15.3	53.5%	1.73	38.2%	153
30	850	98.2	72.1	26.1	176	15.3	51.4%	1.79	35.0%	151

## Supplementary Materials

The following are available online at https://www.mdpi.com/1099-4300/22/4/476/s1. Data that support the values shown in Table 4, Table 5 and Table 6 are available. They include the thermodynamic properties and mass flow rates for all representative points of all configurations at the different operating conditions.

## Nomenclature

*Acronyms*	
CC	Combined cycle
CCGT	Combined cycle gas turbine
CCGT-R-DRDE	CCGT with recuperative GT and DRDE cycle
CSP	Concentrating solar power
DRDE	Double recuperative double expansion
GT	Gas turbine
HP	High pressure
HRSG	Heat recovery steam generator
HRVG	Heat recovery vapor generator
HTF	Heat transfer fluid
ISCC	Integrated solar combined cycle
ISCC-R-DRDE	ISCC with recuperative GT and DRDE cycle
LP	Low pressure
O&M	Operation and maintenance
ORC	Organic Rankine cycle
PTC	Parabolic trough collectors
SSG	Solar steam generator
ST	Steam turbine
SVG	Solar vapor generator
TMY	Typical meteorological year
VT	Vapor turbine
*Symbols*	
*C*	Cost of energy (€·J^−1^)
*DNI*	Direct normal irradiation (W·m^−2^)
*E*	Energy (J)
*h*	Hour (h)
*H_c_*	Lower heating value (J·kg^−1^)
*HR*	Heat rate (-)
*IAM*	Incidence angle modifier (-)
*LC*	Levelized cost (€)
*LCOE*	Levelized cost of energy (€·J^−1^)
*ṁ*	Mass flow rate (kg·s^−1^)
*n*	Yearly frequency (-)
*p*	Pressure (Pa)
*P*	Power (W)
$\dot{Q}\;$	Thermal power (W)
*t*	Time (s)
*T*	Temperature (K)
*U*	Overall heat transfer coefficient (W·K^−1^·m^−2^)
*UA*	Thermal conductance (W·K^−1^)
*Greek letters*	
Δ	Increment
*ε*	Effectiveness (-)
*ξ*	Pressure drop (-)
*η*	Efficiency (-)
*Subscripts*	
*amb*	Ambient
*exh*	Exhaust gas
*f*	Fuel
gt	Gas turbine
inv	Investment
ise	Internal solar-to-electricity
*l*	Length coordinate
*max*	Maximum
R	Recuperator
sol	Solar
st	Steam turbine
tot	Total
